# *Paenibacillus lutrae* sp. nov., A Chitinolytic Species Isolated from A River Otter in Castril Natural Park, Granada, Spain

**DOI:** 10.3390/microorganisms7120637

**Published:** 2019-12-02

**Authors:** Miguel Rodríguez, José Carlos Reina, Victoria Béjar, Inmaculada Llamas

**Affiliations:** 1 Department of Microbiology, Faculty of Pharmacy, University of Granada, 18071 Granada, Spain; miguelrg@correo.ugr.es (M.R.); josecreina@ugr.es (J.C.R.); vbejar@ugr.es (V.B.); 2 Institute of Biotechnology, Biomedical Research Center (CIBM), University of Granada, 18100 Granada, Spain

**Keywords:** *Paenibacillus lutrae*, river otter, new species, chitinolytic activity

## Abstract

A highly chitinolytic facultative anaerobic, chemoheterotrophic, endospore-forming, Gram-stain-positive, rod-shaped bacterial strain N10^T^ was isolated from the feces of a river otter in the Castril Natural Park (Granada, Spain). It is a slightly halophilic, motile, catalase-, oxidase-, ACC deaminase- and C4 and C8 lipase-positive strain. It is aerobic, respiratory and has a fermentative metabolism using oxygen as an electron acceptor, produces acids from glucose and can fix nitrogen. Phylogenetic analysis of the 16S rRNA gene sequence, multilocus sequence analysis (MLSA) of 16S rRNA, *gyrB*, *recA* and *rpoB,* as well as phylogenomic analyses indicate that strain N10^T^ is a novel species of the genus *Paenibacillus,* with the highest 16S rRNA sequence similarity (95.4%) to *P. chitinolyticus* LMG 18047^T^ and <95% similarity to other species of the genus *Paenibacillus*. Digital DNA–DNA hybridization (dDDH) and average nucleotide identity (ANIb) were 21.1% and <75%, respectively. Its major cellular fatty acids were anteiso-C_15:0_, C_16:0_, and iso-C_15:0_. G + C content ranged between 45%–50%. Using 16S rRNA phylogenetic and in silico phylogenomic analyses, together with chemotaxonomic and phenotypic data, we demonstrate that type strain N10^T^ (= CECT 9541^T^ =LMG 30535^T^) is a novel species of genus *Paenibacillus* and the name *Paenibacillus lutrae* sp. nov. is proposed.

## 1. Introduction

Chitins, the second most abundant polymers after cellulose, are widely found in nature. They are present in arthropod, nematode, and mollusc integuments, in insect gut linings, fungal cell walls, some algae and in protozoan cysts [1]. Given their wide distribution, chitin degraders could be used effectively to combat pathogenic organisms, of which chitin is an essential component. Chitin can be degraded by hydrolytic enzymes, called chitinases (EC 3.2.1.14), which are produced by several viruses, as well as prokaryote and eukaryote organisms [2,3].

The principal bacterial groups capable of degrading chitin belong to the genera *Cytophaga*, *Flavobacterium* [4,5], and *Actinomyces* [6,7]. Other groups, which are also active chitin degraders, include species of the genera *Bacillus, Paenibacillus, Serratia, Sporosarcina, Exiguobacterium* [6,7,8,9,10], *Aeromonas* [11], *Pseudomonas* [12], *Stenotrophomonas* [13], *Streptomyces, Arthrobacter* and *Micromonospora* [14,15,16,17,18,19].

Chitin-degrading bacteria mainly inhabit marine habitats, where they account for approximately 1% of all prokaryotes [20,21]. These bacteria can also be isolated from soil and other habitats such as the digestive system of certain mammals and insects [22,23] with chitin digestion is proven to require bacterial symbiosis. The biotechnological applications of chitinolytic microorganisms and their enzymes include chitin bioconversion in fertilizer compounds; in insecticides, nematicides, and fungicides used in agricultural pest control; as well as for biodegradable materials, such as contact lenses, artificial skin, and sutures, in human health care [24,25,26,27].

*Paenibacillus* species were originally assigned to the genus *Bacillus*, first described in 1872, and the type species was *B. subtilis*. A phylogenetic analysis of *Bacillus* species in 1992 based on 16S rRNA sequencing segregated the species into at least five distinct clusters, one of which was reassigned to the novel genus *Paenibacillus* [28]. Members of this genus, currently composed of over two hundred species, have the following characteristics: ellipsoidal spores in swollen sporangia, G + C content ranging from 39 to 54 mol %, 12-methyltetradecanoic acid (anteiso-C_15:0_) as the major cellular fatty acid, and menaquinone-7 (MK-7) as the principal isoprenoid quinone. The chitinolytic species are *P. alvei* [29], *P. chitinolyticus* [30,31], *P. dendritiformis* [32], *P. elgii* [33], *P. larvae* [34], *P. macerans* [35,36], *P. mucilaginosus* [37], *P. pasadenensis* [38], *P. pulvifaciens* [39], *P. timonensis* [40], and *P. xylanilyticus* [41].

Our research group was interested in selecting new chitin-degrading bacteria with potential biotechnological applications. Given that river otters are known to contain *Firmicutes* as the dominant group in their intestinal microbiota [42] and that river crabs are part of their diet, we analyzed the microbiota present in the feces of river otters living in the Castril Natural Park in Granada, Spain. In this study, we describe a new chitin-degrading species isolated from the feces of a river otter and demonstrate that the intestinal microbiota of crustacean-eating otters is a good source of bacteria with biotechnological potential as chitinase producers.

## 2. Material and Methods

### 2.1. Bacterial Strains and Growth Conditions

Strain N10^T^ was isolated from the feces of a river otter in Castril Natural Park, Granada, Spain (37°52′00′′ N 2°45′58′′ W). One gram of feces was suspended in 0.9% (*w*/*v*) saline solution and heated to 80 °C for 20 min to select sporulated bacteria. 0.1 mL of the sample was then plated on Luria–Bertani (LB) agar medium and incubated at 28 °C for 7 days. The different isolated colonies were subsequently plated and purified on the same medium. *Paenibacillus polymyxa* CECT 155^T^ and *Paenibacillus chitinolyticus* LMG 18047^T^ were used as reference strains for comparative purposes. All strains were maintained and cultivated in LB medium at 28 °C unless otherwise stated.

### 2.2. Phenotypic Characterization

Minimal standards for describing new taxa of aerobic, endospore-forming bacteria as recommended by Logan et al. [43] were used in the description of type species *Paenibacillus*. The morphology, size, and pigmentation of colonies were observed on LB agar medium after 48 h of incubation at 28 °C. Cell morphology, flagella type, size, spore morphology and position in the sporangia were determined by transmission electron microscopy (TEM) after negatively staining cells with 2% (*w/v*) uranyl acetate. Motility was observed using log phase culture according to the hanging drop method. Oxidase activity [44] and catalase were also determined.

Growth range and optimum growth were determined in LB broth medium at different NaCl concentrations ranging from 0% to 10% (*w*/*v*) in 1.0 intervals at pH 7. The pH growth range and optimum were determined in LB broth medium. pH was adjusted from 4 to 11 in 1.0 pH unit intervals, using the following buffer systems: 0.1 M citric acid/0.1 M sodium citrate (pH 4.0–5.0,); 0.1 M KH_2_PO_4_/0.1 M NaOH (pH 6.0–8.0); 0.1 M NaHCO_3_/0.1 M Na_2_CO_3_ (pH 9.0–10.0); 0.2 M KH_2_PO_4_/0.1 M NaOH (pH 11.0) [45]. Optical density at 600 nm was monitored using a spectrophotometer to determine bacterial growth. The temperature range and optimum for growth were determined at 15, 22, 28, 37, and 45 °C on LB agar plates. Anaerobic growth capacity was evaluated on LB agar medium by incubation in hermetic jars using the Gas Pak anaerobic system (BBL) to generate an anaerobic atmosphere over a one-week period. H_2_S production was tested in liquid LB medium supplemented with 0.01% (*w*/*v*) L-cysteine using a strip of paper impregnated with lead acetate and placed in the neck of the tube as an indicator [46]. To assay nitrate reduction, 0.2% (*w*/*v*) KNO_3_ was added to the LB medium. Nitrite was detected with α-naphthylamine/sulfanilic acid reagents, and gas production was detected in inverted Durham tubes [47]. Indole production was determined using peptone broth as the growth medium [48], while the methyl-red and Voges–Proskauer tests used peptone supplemented with 0.5% *w*/*v* glucose [49]. The use of citrate as sole carbon source was tested in Simmons medium [50].

Hydrolysis of chitin, casein, cellulose, DNA, Tweens^®^ 20 and 80; and production of acid and alkaline phosphatase, lecithinase, lipase, and hemolysine; and the liquefaction of gelatine were also performed [50]. Growth on MacConkey agar medium was assessed after 7 days of incubation. Nitrogen-fixing capacity was tested in Burk medium by following the protocol described by Stella and Suhaimi [51]. The 1-aminocyclopropane-1-carboxylic acid (ACC) deaminase enzyme was detected according to the protocol described by Poonguzhali et al. [52]. Siderophore production was tested using the chromeazurol sulphonate (CAS) method [53]. Other biochemical characteristics were analyzed using API 50CH, API 20NE and API ZYM, according to the manufacturer’s instructions.

The diffusion agar method was used to assay antimicrobial susceptibility [54]. The following antimicrobial discs were tested: ampicillin 10 μg, chloramphenicol 30 μg, kanamycin 30 μg, nalidixic acid 30 μg, rifampicin 2 μg, streptomycin 10 μg, gentamicin 10 μg, neomycin 30 μg, novobiocin 30 μg, penicillin G 10 μg, tetracyclin 30 μg, and trimethoprim/sulfamethoxazole 1.25/23.75 μg.

### 2.3. Chemotaxonomic Analysis

The cellular fatty acids, polar lipids, respiratory quinones and the diamino acid of the cell-wall peptidoglycan were analyzed at the German Collection of Microorganisms and Cell Cultures (DSMZ) according to the protocol described by Tindall et al. [55], Tindall [56,57] and Schumann [58], respectively. For these analyses, the cell mass of N10^T^ strain, *P. chitinolyticus* LMG 18047^T^ and *P. polymyxa* CECT 155^T^, was obtained after growing 24 h at 28 °C in TSB medium.

### 2.4. Phylogenetic 16S rRNA Gene Analysis

Genomic DNA was isolated by using the X-DNA purification kit (Xtrem Biotech S.L., Granada, Spain). The 16S rRNA gene was amplified with the aid of universal bacterial primers 16F27 and 16R1488. The PCR product was purified and cloned into the pGEM^®^-T vector (Promega, Wisconsin, USA). Direct sequencing of PCR-amplified DNA was determined using an ABI PRISM DyeTerminator Cycle Sequencing Ready Reaction kit (Perkin-Elmer, Massachusetts, USA) and an ABI PRISM 377 sequencer (Perkin-Elmer) according to the manufacturer’s instructions. The DNA sequence obtained was compared to reference 16S rRNA gene sequences available in GenBank and EMBL databases obtained from the NCBI Genome Database using BLASTN software [59] and the EzBioCloud server (https://www.ezbiocould.net) [60]. The phylogenetic analysis was carried out using Molecular Evolutionary Genetics Analysis (MEGA) software version X [61], following multiple data alignments by CLUSTAL OMEGA [62]. Distances and clustering were determined according to the neighbor-joining and maximum-parsimony methods. Cluster stability was ascertained by bootstrap analysis (1000 replications). The GenBank/EMBL/DDBJ accession number for the 16S rRNA sequence of *Paenibacillus lutrae* N10^T^ is MG831947.

### 2.5. G+C Content

DNA was purified according to Marmur’s method [63]. G+C content of *Paenibacillus* spp. was determined by the thermal denaturalization (T_m_) method using *Escherichia coli* NCTC 9001 as control [64].

### 2.6. Genome Sequencing and Assembly

DNA of strain N10^T^, previously extracted according to the protocol described by Marmur [63], was sequenced using the Illumina Hi-Seq platform at the STAB VIDA facility (Caparica, Portugal) with 2 × 150-bp paired-end reads. The reads, which were processed by BBDuk (https://sourceforge.net/projects/bbmap/) to remove adapters and low-quality bases and reads, were then assembled using SPAdes software v. 3.11.1 [65] and the contigs were blasted against the nr/nt database to remove those contigs belonging to contaminants.

### 2.7. In Silico ANI and DDH

Average nucleotide identity based on BLAST (ANIb) and MUMmer (ANIm) algorithms was determined with the aid of JSpeciesWS software [66], while digital DNA–DNA hybridization (dDDH) was calculated using the BLAST+ algorithm on the DSMZ Genome-to-Genome Distance Calculator (GGDC 2.1) platform [67]. The results presented in this study are based on the recommended Formula 2 (identities/HSP length), which, being independent of genome length, is robustly protected against the use of incomplete draft genomes. OrthoANI was similarly calculated using OrthoANI software [68].

### 2.8. Analysis of Multilocus Sequences and Core Orthologous Genes

A phylogenetic tree was built by aligning the concatenated sequences of the 16S rRNA, *gyrB*, *recA,* and *rpoB* genes obtained with the aid of CLUSTAL OMEGA [62]. For strain N10^T^, these genes were extracted from the annotation made with Rapid Annotation Subsystem Technology (RAST) software [69], whereas for the rest of the species, the sequences were obtained from their publicly available genome deposited in NCBI. Finally, the phylogenetic tree was constructed using iQ-TREE 1.6.12 [70] according to the maximum likelihood method, taking into account the partitions of genes and codons [71,72], with *Bacillus subtilis* subsp. *subtilis* ATCC 6051^T^ as the outgroup. A core genome analysis of strain N10^T^ and the five closest related bacteria based on their 16s rRNA percentage of similarity for which their genome was available was also performed using Bacterial Pan Genome Analysis (BPGA) software [73] with the default parameters. After obtaining the core of these six bacterial genomes, all protein orthologs belonging to the core genome were concatenated and aligned by MAFFT [74]. A phylogenomic tree of the core genes of the species was then constructed using MEGA X software according to the neighbor-joining method.

## 3. Results and Discussion

### 3.1. Phenotypic Characterization

**Figure 1:**
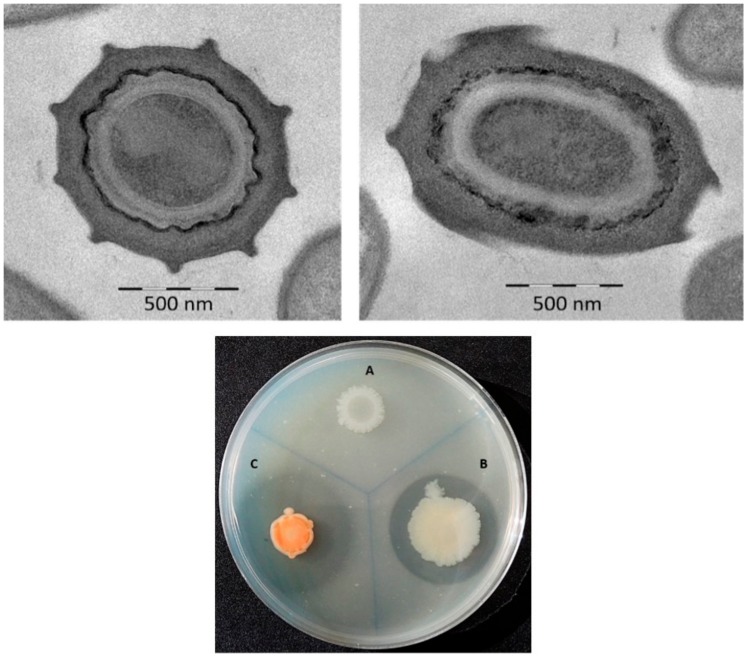
Transmission electron micrographs of oval-shaped endospores of strain N10^T^ (upper images) and chitinolytic activity (lower image). *P. polymyxa* CECT 155^T^ (**A**), *P. chitinolitycus* LMG 18047^T^ (**B**), and strain N10^T^ (**C**) on LB medium supplemented with 1% (*w*/*v*) colloidal chitin.

Given its chitinolytic activity, strain N10^T^ was selected from among the eleven sporulated strains isolated from the river otter feces and then characterized taxonomically. Its colonies on LB medium had a circular (diameter 1.0–2.0 mm) and convex morphology with a brilliant pink pigmentation after 48 h of incubation. Cells were found to be rod-shaped (2.2 × 0.8 µm), motile by peritrichous flagella and Gram-, catalase- and oxidase-positive. Strain N10^T^ was slightly halophilic and produced oval endospores (1.2 × 0.8 µm) in swollen sporangia (Figure 1).

Strain N10^T^ was found to be a facultative anaerobe, with no differences between aerobic and anaerobic growth being detected. It grew in a 15 to 37 °C temperature range, with 28 °C as optimum, in a pH range of 7 to 10, with 8 pH as optimum. The strain, which grew in the presence of 0% to 3% (*w*/*v*) concentrations of NaCl, with optimum growth at 1% (*w*/*v*) NaCl, was slightly halophilic.

Strain N10^T^ was found to produce H_2_S from L-cystein; to hydrolyze chitin (Figure 1) and Tween^®^ 20, but not Tween^®^ 80, gelatine, casein, starch, DNA or lecithovitellin; to fix N_2_; and to degrade 1-aminocyclopropane-1-carboxilic acid (ACC). The tests for methyl red, indole, Voges–Proskauer, citrate, hemolysis, alkaline phosphatase, and aerobic nitrate reduction to nitrite, were negative. Its phenotypic characteristics are shown in Table 1 and the species description section. The differential characteristics of strain N10^T^ with respect to the most closely related species, *P. chitinolyticus* (LMG 18047^T^) and with the type strain of genus *P. polymyxa* (CECT 155^T^) are also shown in Table 1. The strain N10^T^ mainly differs in relation to the following features: pink coloration of the colonies, the slight requirements of NaCl, the inability to reduce aerobically nitrate, produce acids from glucose and many other sugar compounds and enzymes such as acid phosphatase, β-galactosidase or β-glucosidase.

### 3.2. Phylogenetic Analysis Based on the 16S rRNA Gene Sequence

**Figure 2:**
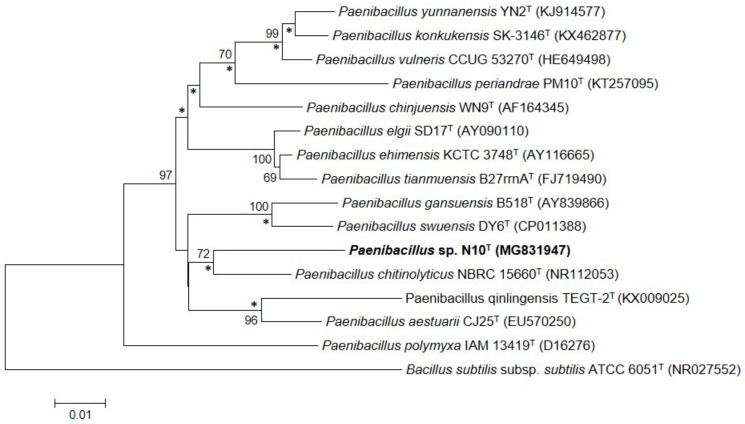
Phylogenetic position of strain N10^T^ based on the neighbor-joining algorithm of the 16S rRNA gene sequence and its relationship with other related species. The GenBank/EMBL/DDBJ accession number of each sequence is shown in parenthesis. Bootstrap values are expressed as percentages of 1000 replications, and those greater than 60% are shown at branch points. Bar shows sequence divergence. Bar, 0.01 substitutions per nucleotide position. Asterisks indicate that the corresponding nodes were also obtained in the trees generated with the maximum-likelihood and maximum-parsimony algorithms.

Sequencing of the cloned 16S rRNA gene of strain N10^T^ resulted in a virtually complete 1520 bp-long sequence. Phylogenetic analysis of the 16S rRNA gene sequences of strain N10^T^ and other 100 related strains revealed that strain N10^T^ is a member of the genus *Paenibacillus* (Figure S1). It showed the highest sequence similarity to *P. chitinolyticus* LMG 18047^T^ (95.4%) and less than 95% similarity to other species of genus *Paenibacillus*. The phylogenetic tree constructed using the neighbor-joining algorithm indicates that strain N10^T^ forms a cluster with the *P. chitinolyticus* species, which showed the highest sequence similarity (Figure 2 and Figure S1). Using maximum-likelihood and maximum-parsimony algorithms, the *Paenibacillus* species was found to have the same phylogenetic distribution (Figures S2 and S3).

### 3.3. Analysis of Cellular Fatty Acids, Polar Lipids, Isoprenoid Quinones and Peptidoglycan Structure

The fatty acid profile of strain N10^T^, which showed a predominance of anteiso-C_15:0_ (56.95%), C_16:0_ (11.69%), and iso-C_15:0_ (8.46%), was similar from that of the most closely related strains and the type strain of the genus (Table 2). The major isoprenoid quinone of strain N10^T^ was found to be MK-7, while diphosphatidylglycerols (DPGs), phosphatidylglycerol (PG), phosphatidylethanolamine (PE), and phosphoaminolipids (PNL) were the main polar lipids, features also commonly found in genus *Paenibacillus* [75]. The results of two-dimensional thin-layer chromatography (TLC) analysis of polar lipids are shown in the Supplementary Materials (Figure S4). The diamino acid of the cell-wall peptidoglycan for N10^T^ strain and *P. chitinolyticus* LMG 18047^T^ was *meso*-diamino-pimelic acid (type A1γ), as found in other members of *Paenibacillus* genus.

### 3.4. G+C Content

The G+C content of strain N10^T^, determined by the melting temperature (*T_m_*) method, was in the 45–48 mol% range, while an in silico analysis of its draft genome G+C content produced a value of 49.8 mol%. These percentages fall within the broad 39 to 54 mol% range commonly accepted for members of genus *Paenibacillus* [28].

### 3.5. Whole-Genome Sequencing and Assembly

The over 5.54 Mbp-long draft genome of strain N10^T^, after manual curation, resulted in 48 contigs, while the 6.43 Mbp-long draft genome of *P. chitinolyticus* LMG 18047^T^ in the public databases contains 32 contigs. After assembling the whole genome of strain N10^T^, the sequence was tested using Quality Assessment Tool for Genome Assemblies (QUAST) software. The results indicate that the whole-genome sequence was of sufficient quality, with an N50 value of 286655, an L50 value of 5, and 380X coverage. PGAP annotation [76] showed a total of 4718 protein-coding genes (PCGs), 3771 of which were assigned to a functional COG category in the EggNOG database [77,78]; categories K (transcription) and G (carbohydrate transport and metabolism) were the most abundant, with seven chitinase genes being found in the latter category. Strain N10^T^ presented 117 RNAs and 8 16S rRNA gene copies. Nevertheless, only one of them was complete, whereas the rest of the copies corresponded to partial sequences. This genome sequence, which was deposited in the GenBank/EMBL/DDBJ database under accession number RHLK00000000, was used for further analysis.

### 3.6. ANI and dDDH Calculations

The average nucleotide identities (ANIs), based on BLAST (ANIb) and MUMmer (ANIm), for strain N10^T^ and related *Paenibacillus* species, are shown in Table S1. The ANIb and ANIm values between strain N10^T^ and the most closely related *P. chitinolyticus* LMG 18047^T^ were 74.4% and 84.6%, respectively. In all cases, the ANI values for strain N10^T^ and all species studied were lower than the cut-off for species demarcation (95%–96%) proposed by Richter and Rosselló-Móra [79,80]. Although not clear genus delimitation has been elucidated based on ANI values [81], Pérez-Cataluña et al. [74] consider that 68% could be the genus ANI threshold value. The ANIb between N10^T^ and *P. chitinolyticus* LMG 18047^T^ is over this value, reinforcing that strain N10^T^ belongs to the *Paenibacillus* genus. As expected, according to Lee et al. [68], the OrthoANI value between N10^T^ strain and *P. chitinolyticus* was slightly higher (75.6%) than the ANIb value (74.4%) (Table S2).

A comparison of the whole-genome sequences of the species analyzed showed digital DNA–DNA hybridization (dDDH) similarity of less than 32% (Table S2). The dDDH similarity between strain N10^T^ and *P. chitinolyticus* LMG 18047^T^ was 21.1%, a percentage that supports species delineation, as the established cut-off value for dDDH is 70% [82]. This was also the case for dDDH similarity between N10^T^ and P. *polymyxa,* and the closest related species *P. elgii, P. vulneris* and *P. qinlingensis,* with percentages of 31.4%, 19.9%, 21.6% and 19.3%, respectively.

### 3.7. Phylogenetic Analysis of Multilocus Sequences and Core Orthologous Proteins

**Figure 3:**
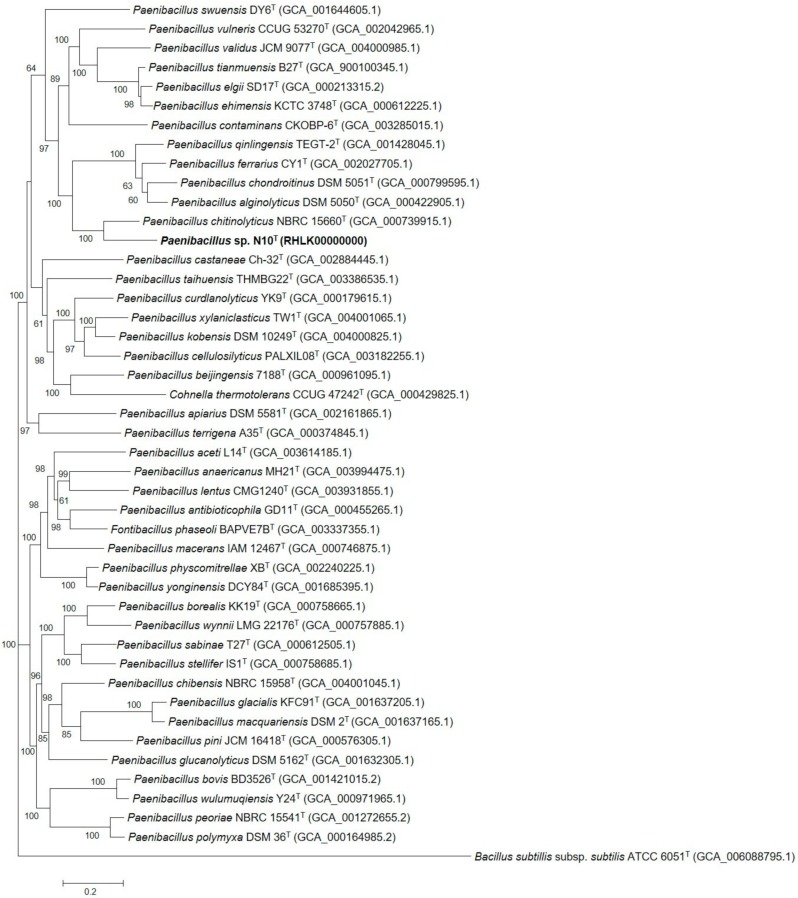
Maximum-likelihood tree based on a comparison of the concatenated 16S rRNA, *gyrB, recA,* and *rpoB* gene sequences, showing the phylogenetic position of strain N10^T^ and its relationship with the 43 closely related species of genus *Paenibacillus*. Bootstrap values are expressed as percentages of 1000 replications, and those greater than 60% are shown at branch points. Bar, 0.2 substitutions per nucleotide position.

For a more robust analysis, a phylogenetic tree was built by aligning the concatenated sequences of the 16S rRNA and the three housekeeping *gyrB*, *recA,* and *rpoB* genes resulting in an alignment of 8269 nt. The 43 type strains out of the 102 closest species for which these sequences were available and correctly annotated, along with N10^T^ and *Bacillus subtilis* subsp. *subtilis* ATCC 6051^T^ as the outgroup, were used for this analysis. The resulting phylogenetic tree constructed according to the maximum likelihood method (Figure 3) confirms that strain N10^T^ belongs to the genus *Paenibacillus* in accordance with the result obtained in the previous phylogenetic analysis using 16S rRNA sequences. The tree was carried out taking into account the partitions and codons of each gene.

**Figure 4:**
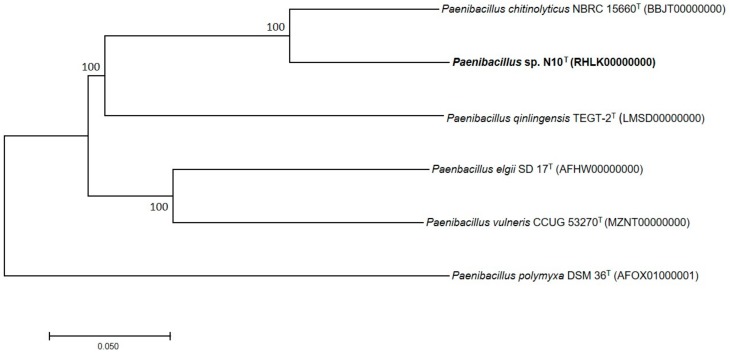
Tree constructed according to the neighbor-joining method based on 1078 core orthologous proteins of strain N10^T^ and five additional most closely related species of genus *Paenibacillus*. Bootstrap values are expressed as percentages of 1000 replications, and those over 60% are shown at branch points. Bar, 0.05 substitutions per nucleotide position.

A phylogenetic tree reconstruction based on the concatenated alignment of the 1078 core orthologous proteins confirmed that strain N10^T^ and the five closest related bacteria have the same phylogenetic distribution (Figure 4), thus corroborating our previous results.

## 4. Conclusions

The polyphasic taxonomic study, which included a phenotypic and phylogenetic 16S rRNA, *gyrB, recA*, and *rpoB* gene analysis, as well as chemotaxonomic and genomic analyses, showed that strain N10^T^ isolated from river otter (*L. lutra)* feces constitutes a novel species (proposed name: *Paenibacillus lutrae* sp. nov.) within genus *Paenibacillus,* with type strain N10^T^ (= CECT 9541^T^ = LMG 30535^T^). The description of *Paenibacillus lutrae* sp. nov. is summarized in Appendix A.

## Figures and Tables

**Table 1:** Differential characteristics between N10^T^ strain with respect to its closest relative and the type strain of the genus *Paenibacillus.*

Characteristic	1	2	3
Colony Pigmentation	Pink	Cream	Cream-White
NaCl range (%) (*w*/*v*)	0–3	0–4	0–5
NaCl optimum (%) (*w*/*v*)	1	0	0
pH range	7–10	6–9	6–9
pH optimum	8	7	7
Oxidase	+	v	-
Methyl red	-	-	+
Voges-Proskauer	-	-	+
Aerobic nitrate reduction	-	+	+
Hydrolysis of:			
Gelatine	-	v	-
Chitin	+	+	-
Starch	-	-	+
Casein	-	v	+
Tween 80	-	-	+
ACC deaminase	+	+	-
Assimilation of:			
l-arabinose	-	-	+
d-mannose	-	+	+
d-mannitol	-	-	+
*N*-acetyl-glucosamine	-	+	-
Potassium gluconate	-	+	+
Enzymes:			
Alkaline phosphatase	-	+	-
Acid phosphatase	-	+	+
α-galactosidase	-	-	+
β-galactosidase	-	+	+
β-glucosidase	-	+	+
Acids from carbohydrates:			
Glycerol	-	+	+
Galactose	-	+	+
Manose	-	+	+
Amygdaline	-	+	+
Arbutin	-	+	+
Salicin	-	+	+
Cellobiose	-	+	+
Lactose	-	+	+
Melibiose	-	+	+
Sucrose	-	+	+
Trehalose	-	+	+
Gentiobiose	-	+	+
d-turanose	-	+	+
Antimicrobial susceptibility:			
Ampicilin	S	I	S
Cloramphenicol	S	I	S
Kanamycin	R	R	S
Nalidixic acid	S	R	S
Rifampicin	S	S	R
Trimethoprim/sulfamethoxazole	R	S	S
DNA G + C content (Tm) (mol %)	45–48	51–53	43–46
DNA G + C content (*in silico*) (mol %)	49.8	52.4	45.5

Strains: 1, N10^T^ strain; 2, *P. chitinolitycus* LMG 18047^T^; 3, *P. polymyxa* CECT 155^T^. +, positive; -, negative; v, variable; S, susceptible; I, intermediate; R, resistant. All strains were rods, Gram-positive, growth in anaerobic conditions and produce oval spores. All data shown in this table derive from this study. All strains were negative for indole, citrate, cellulase, DNase, lecithinase, hemolysis, and siderophore production and did not grow on MacConkey agar, but were positive for catalase, H_2_S production, nitrogen-fixing and Tween^®^ 20 hydrolysis. The three strains analyzed were negative for arginine dihydrolase (ADH), urease, gelatinase, capric acid, malic acid, adipic acid and phenylacetic acid assimilations, C14 lipase, leucine arylamidase, valine arylamidase, cysteine arylamidase, trypsin, α-chymotrypsin, β-glucuronidase, *N*-acetyl-β-glucosaminidase, α-mannosidase, and α-fucosidase. All strains showed positive results for esculin, d-glucose and d-maltose assimilations, C4 and C8 lipases, α-glucosidase, and naphthol-AS-BI-phosphohydrolase. All strains were positive for acid production from esculin, glucose, glycogen, d-maltose, 1-methyl-α-d-glucopyranoside, starch, and D-ribose. By contrast, they were negative for acid production from d-adonitol, d-arabinose, d- and l-arabitol, dulcitol, erythritol, d- and l-fucose, inositol, 1-methyl-α-d-mannopyranoside, potassium gluconate, potassium 2-ketogluconate, and potassium 5-ketogluconate, d-sorbitol, l-sorbose, d-tagatose, xylitol and d- and l-xylose. All strains were sensitive to gentamicin, neomycin, novobiocin, penicillin G, and tetracycline, but were resistant to streptomycin.

**Table 2:** Cellular fatty acids content of N10^T^ and related species of the genus. Strains: 1, N10^T^; 2, *P.* *chitinolyticus* LMG 18047^T^; 3, *P. polymyxa* CECT 155^T^. -, not detected or lower than 1% of total composition.

Cellular Fatty Acids	1	2	3
Straight-chain fatty acids:			
C_14:0_	2.50	2.49	-
C_15:0_	1.17	-	-
C_16:0_	11.69	25.88	8.39
Branched-chain fatty acids:			
iso-c_14:0_	1.86	-	3.16
iso-c_15:0_	8.46	6.26	3.44
anteiso-c_15:0_	56.95	49.20	47.79
iso-c_16:0_	5.98	2.34	18.80
iso-c_17:0_	2.63	4.73	3.38
anteiso-c_17:0_	5.82	6.76	12.24
Unsaturated fatty acids:			
C_16:1_ ω11c	1.59	-	-

## Supplementary Materials

The following are available online at https://www.mdpi.com/2076-2607/7/12/637/s1, Figure S1: Phylogenetic position of strain N10T based on the neighbour-joining algorithm of the 16S rRNA gene sequence and its relationship with other 100 related species. The GenBank/EMBL/DDBJ accession number of each sequence is shown in parenthesis. Bootstrap values are expressed as percentages of 1000 replications, and those greater than 60% are shown at branch points. Bar shows sequence divergence. Bar, 0.015 substitutions per nucleotide position. Figure S2: Molecular phylogenetic analysis of the 16S rRNA sequence according to the maximum likelihood method. Evolutionary history was inferred using the aforementioned method based on the Jukes-Cantor model. The tree with the highest log likelihood (-5308.40532.87) is shown. Bootstrap values are expressed as percentages of 1000 replications, and those over 60% are shown at branch points. The *Bacillus subtilis* subsp. *subtilis* ATCC 6051^T^ sequence was used as the outgroup. Figure S3: Molecular phylogenetic analysis of the 16S rRNA sequence using the maximum parsimony method. The most parsimonious tree (length = 592) is shown. The consistency, retention and composite indices are 0.501114, 0.554672 and 0.344796 (0.277954), respectively, for all sites and parsimony-informative sites (in parentheses). Bootstrap values are expressed as percentages of 1000 replications, and those over 60% are shown at branch points. The *Bacillus subtilis* subsp. *subtilis* ATCC 6051^T^ sequence was used as the outgroup. Figure S4. Polar lipid profile of strain N10^T^ determined after two-dimensional TLC using molybdatophosphoric acid. AL: aminolipid; DPG: diphosphatidylglycerol; L: lipid; PE: phosphatidylethanolamine; PG: phosphatidylglycerol; PL: phospholipid; PNL: phosphoaminolipid. Table S1. ANIb and ANIm (in brackets) values among the genomes of strain N10^T^ (1) and the most related species of *Paenibacillus* genus: *P. chitinolyticus* LMG18047^T^ (2), *P. polymyxa* CECT155^T^ (3), *P. elgii* SD17^T^ (4), *P. vulneris* CCUG 53270^T^ (5) and *P. qinlingensis* TEGT-2^T^ (6). Table S2. Genome sequence similarity between N10^T^ strain and genome sequences of closely related type strains with available genomes.

## Appendix A

### Description of Paenibacillus lutrae sp. nov

*Paenibacillus lutrae* (lu’trae. L. fem. gen. n. *lutrae* pertaining to the *Lutra lutra* otter from which the bacterium was isolated).

*Paenibacillus lutrae* sp. nov. is a motile, straight, Gram-positive, 2.2 × 0.8 µm rod-shaped bacteria which forms oval endospores in swollen sporangia. The colonies of *P. lutrae* on LB medium are a pink color after growing for 48 h at 28 °C. Cells growth pattern is uniform in a liquid medium. They are capable of growing in the presence of 0% to 3% (*w*/*v*) NaCl concentrations, with 1% (*w*/*v*) as optimum. The cells grow in a temperature range of 15 to 37 °C, with optimum growth at 28 °C, and a pH range of 7 to 10, with pH 8 as optimum. *P. lutrae* is a chemoorganotrophic and anaerobic facultative microorganism. Under aerobic conditions, nitrate is not reduced. Indole and methyl-red tests are negative, and catalase and oxidase are negative. Acids are not produced from adonitol, d-celobiose, d-fructose, d-galactose, *myo*-inositol, lactose, d-mannitol, d-mannose, d-melezitose, l-rhamnose, sucrose, d-salicilin, d-sorbitol, sorbose or d-trehalose. Tween 20 is hydrolyzed but, starch, casein, lecithovitellin, gelatine, Tween 80, urea, blood, or phosphates are not. H_2_S is produced from L-cysteine but not butanediol from glucose (Voges–Proskauer negative). ONPG is negative. No growth occurs on MacConkey agar. They are susceptible to ampicillin (10 μg), chloramphenicol (30 μg), gentamycin (10 μg), neomycin (30 μg), novobiocin (30 μg), penicillin G (10 μg), nalidixic acid (30 μg), rifampicin (2 μg) and tetracycline (30 μg). They are resistant to streptomycin (10 μg), kanamycin (30 μg) and trimetroprim/sulphametoxazol (1.25/23.75 μg).

The principal fatty acids of *P. lutrae* are anteiso-C_15:0_ (56.95%), C_16:0_ (11.69%), and iso-C_15:0_ (8.46%), and the main isoprenoid quinone is MK-7. The principal polar lipids are diphosphatidylglycerols (DPGs), phosphatidylglycerol (PG), phosphatidylethanolamine (PE), and phosphoaminolipids (PNL). The diamino acid of the cell-wall peptidoglycan was *meso*-diamino-pimelic acid (type A1γ). DNA G+C content was in the 45–48 mol% range according to the *T_m_* method and 49.8 mol% according to in silico determination.

Type strain N10^T^ (= CECT 9541^T^ = LMG 30535^T^) was isolated from the feces of a river otter in Castril Natural Park in Granada (Spain).

The GenBank/EMBL/DDBJ accession number for the 16S rRNA sequence of *Paenibacillus lutrae* N10^T^ is MG831947, and the complete genome is deposited under the accession number RHLK00000000.
