# Supplementary material for: Paenibacillus lutrae sp. nov., A Chitinolytic Species Isolated from A River Otter in Castril Natural Park, Granada, Spain
Source: Microorganisms. 2019 Dec 2;7(12):637. doi: 10.3390/microorganisms7120637 (PMC6955709; doi:10.3390/microorganisms7120637)
Supplement: Supplementary file 1 [file microorganisms-07-00637-s001.pdf]

***Paenibacillus lutrae* sp. nov., a chitinolytic species isolated from a river otter in Castril Natural Park, Granada, Spain**

Miguel Rodríguez,[1,2] José Carlos Reina,[1] Victoria Béjar,[1,2] Inmaculada Llamas[1,2*]

[1]*Department of Microbiology, Faculty of Pharmacy, University of Granada, Granada, Spain*

[2]*Institute of Biotechnology, Biomedical Research Center (CIBM), University of Granada, Granada, Spain*

*Corresponding author:

I. Llamas; e-mail address: illamas@ugr.es

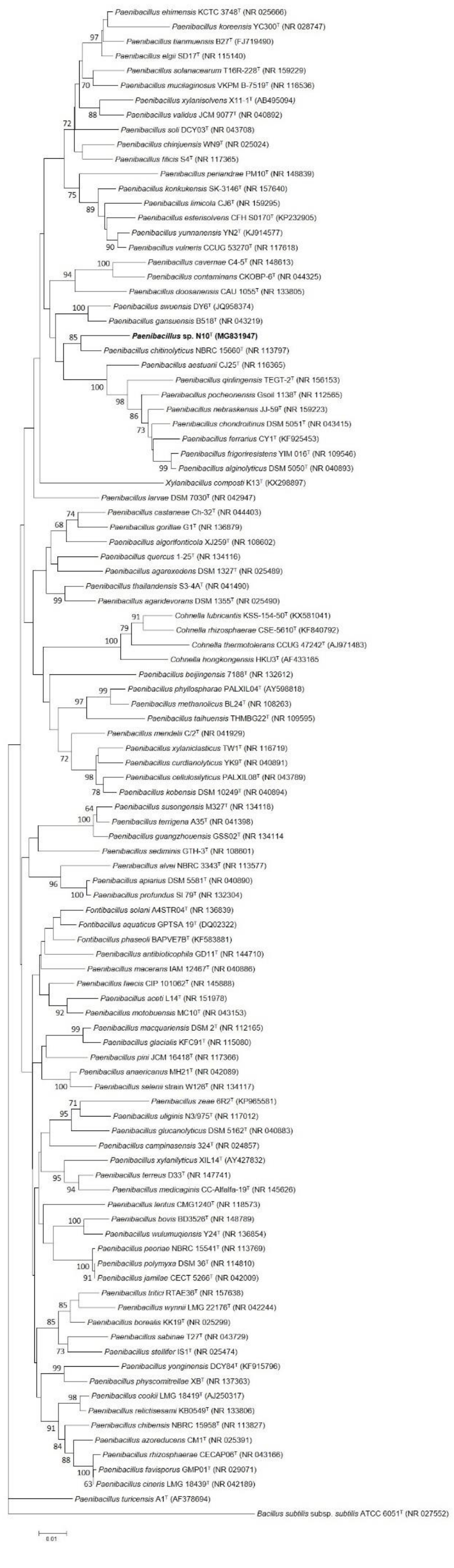
Paenibacillus ehimensis KCTC 3748^{T} (NR 025666)
Paenibacillus koreensis YC300^{T} (NR 028747)
Paenibacillus tianmuensis B27^{T} (FJ719490)
Paenibacillus elgii SD17^{T} (NR 115140)
Paenibacillus solanacearum T16R-228^{T} (NR 159229)
Paenibacillus mucilaginosus VKPM B-7519^{T} (NR 116536)
Paenibacillus xylanisolvens X11-1^{T} (AB495094)
Paenibacillus validus JCM 9077^{T} (NR 040892)
Paenibacillus soli DCY03^{T} (NR 043708)
Paenibacillus chinjuensis WN9^{T} (NR 025024)
Paenibacillus ficus S4^{T} (NR 117365)
Paenibacillus periandrae PM10^{T} (NR 148839)
Paenibacillus konkukensis SK-3146^{T} (NR 157640)
Paenibacillus limicola CJ6^{T} (NR 159295)
Paenibacillus esterisolvens CFH S0170^{T} (KP232905)
Paenibacillus yunnanensis YN2^{T} (KJ914577)
Paenibacillus vulneris CCUG 53270^{T} (NR 117618)
Paenibacillus cavernae C4-5^{T} (NR 148613)
Paenibacillus contaminans CKOBP-6^{T} (NR 044325)
Paenibacillus doosanensis CAU 1055^{T} (NR 133805)
Paenibacillus swuensis DY6^{T} (JQ958374)
Paenibacillus gansuensis B518^{T} (NR 043219)
Paenibacillus sp. N10^{T} (MG831947)
Paenibacillus chitinolyticus NBRC 15660^{T} (NR 113797)
Paenibacillus aestuarii CJ25^{T} (NR 116365)
Paenibacillus qinlingensis TEGT-2^{T} (NR 156153)
Paenibacillus pocheonensis Gsoil 1138^{T} (NR 112565)
Paenibacillus nebraskensis JJ-59^{T} (NR 159223)
Paenibacillus chondroitinus DSM 5051^{T} (NR 043415)
Paenibacillus ferrarius CY1^{T} (KF925453)
Paenibacillus frigoriresistens YIM 016^{T} (NR 109546)
Paenibacillus alginolyticus DSM 5050^{T} (NR 040893)
Xylanibacillus composti K13^{T} (KX298897)
Paenibacillus larvae DSM 7030^{T} (NR 042947)
Paenibacillus castaneae Ch-32^{T} (NR 044403)
Paenibacillus gorillae G1^{T} (NR 136879)
Paenibacillus algorifonticola XJ259^{T} (NR 108602)
Paenibacillus quercus 1-25^{T} (NR 134116)
Paenibacillus agarexedens DSM 1327^{T} (NR 025489)
Paenibacillus thailandensis S3-4A^{T} (NR 041490)
Paenibacillus agaridevorans DSM 1355^{T} (NR 025490)
Cohnella lubricantis KSS-154-50^{T} (KX581041)
Cohnella rhizosphaerae CSE-5610^{T} (KF840792)
Cohnella thermotolerans CCUG 47242^{T} (AJ971483)
Cohnella hongkongensis HKU3^{T} (AF433165)
Paenibacillus beijingensis 7188^{T} (NR 132612)
Paenibacillus phyllosphaerae PALXIL04^{T} (AY598818)
Paenibacillus methanolicus BL24^{T} (NR 108263)
Paenibacillus taihuensis THMBG22^{T} (NR 109595)
Paenibacillus mendelii C/2^{T} (NR 041929)
Paenibacillus xylaniclasticus TW1^{T} (NR 116719)
Paenibacillus curdlanolyticus YK9^{T} (NR 040891)
Paenibacillus cellulosilyticus PALXIL08^{T} (NR 043789)
Paenibacillus kobensis DSM 10249^{T} (NR 040894)
Paenibacillus susongensis M327^{T} (NR 134118)
Paenibacillus terrigena A35^{T} (NR 041398)
Paenibacillus guangzhouensis GSS02^{T} (NR 134114)
Paenibacillus sediminis GTH-3^{T} (NR 108601)
Paenibacillus alvei NBRC 3343^{T} (NR 113577)
Paenibacillus apiarius DSM 5581^{T} (NR 040890)
Paenibacillus profundus Sl 79^{T} (NR 132304)
Fontibacillus solani A4STR04^{T} (NR 136839)
Fontibacillus aquaticus GPTSA 19^{T} (DQ02322)
Fontibacillus phaseoli BAPVE7B^{T} (KF583881)
Paenibacillus antibioticophila GD11^{T} (NR 144710)
Paenibacillus macerans IAM 12467^{T} (NR 040886)
Paenibacillus faecis CIP 101062^{T} (NR 145888)
Paenibacillus aceti L14^{T} (NR 151978)
Paenibacillus motobuensis MC10^{T} (NR 043153)
Paenibacillus macquariensis DSM 2^{T} (NR 112165)
Paenibacillus glacialis KFC91^{T} (NR 115080)
Paenibacillus pini JCM 16418^{T} (NR 117366)
Paenibacillus anaericanus MH21^{T} (NR 042089)
Paenibacillus selenii strain W126^{T} (NR 134117)
Paenibacillus zeae 6R2^{T} (KP965581)
Paenibacillus uliginis N3/975^{T} (NR 117012)
Paenibacillus glucanolyticus DSM 5162^{T} (NR 040883)
Paenibacillus campinasensis 324^{T} (NR 024857)
Paenibacillus xylanilyticus XIL14^{T} (AY427832)
Paenibacillus terreus D33^{T} (NR 147741)
Paenibacillus medicaginis CC-Alfalfa-19^{T} (NR 145626)
Paenibacillus lentus CMG1240^{T} (NR 118573)
Paenibacillus bovis BD3526^{T} (NR 148789)
Paenibacillus wulumuqiensis Y24^{T} (NR 136854)
Paenibacillus peoriae NBRC 15541^{T} (NR 113769)
Paenibacillus polymyxa DSM 36^{T} (NR 114810)
Paenibacillus jamilae CECT 5266^{T} (NR 042009)
Paenibacillus tritici RTAE36^{T} (NR 157638)
Paenibacillus wynnii LMG 22176^{T} (NR 042244)
Paenibacillus borealis KK19^{T} (NR 025299)
Paenibacillus sabinae T27^{T} (NR 043729)
Paenibacillus stellifer IS1^{T} (NR 025474)
Paenibacillus yonginensis DCY84^{T} (KF915796)
Paenibacillus physcomitrellae XB^{T} (NR 137363)
Paenibacillus cookii LMG 18419^{T} (AJ250317)
Paenibacillus relictisesami KB0549^{T} (NR 133806)
Paenibacillus chibensis NBRC 15958^{T} (NR 113827)
Paenibacillus azoreducens CM1^{T} (NR 025391)
Paenibacillus rhizosphaerae CECAP06^{T} (NR 043166)
Paenibacillus favisporus GMP01^{T} (NR 029071)
Paenibacillus cineris LMG 18439^{T} (NR 042189)
Paenibacillus turicensis A1^{T} (AF378694)
Bacillus subtilis subsp. subtilis ATCC 6051^{T} (NR 027552)
0.01

Figure S1: Phylogenetic position of strain N10^{T} based on the neighbor-joining algorithm of the 16S rRNA gene sequence and its relationship with other 100 related species. The GenBank/EMBL/DDBJ accession number of each sequence is shown in parenthesis. Bootstrap values are expressed as percentages of 1,000 replications, and those greater than 60% are shown at branch points. Bar shows sequence divergence. Bar, 0.01 substitutions per nucleotide position.

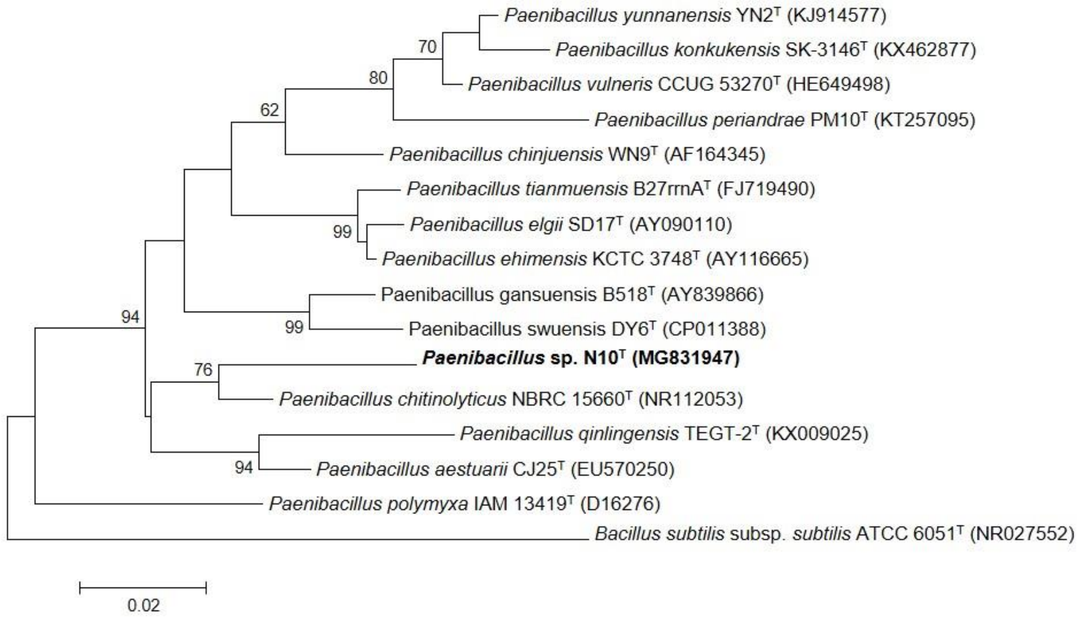

Figure S2: Molecular phylogenetic analysis of the 16S rRNA sequence according to the maximum likelihood method. Evolutionary history was inferred using the aforementioned method based on the Jukes-Cantor model. The tree with the highest log likelihood (-5308.40) is shown. Bootstrap values are expressed as percentages of 1,000 replications, and those over 60% are shown at branch points. The *Bacillus subtilis* subsp. *subtilis* ATCC 6051^{T} sequence was used as the outgroup.

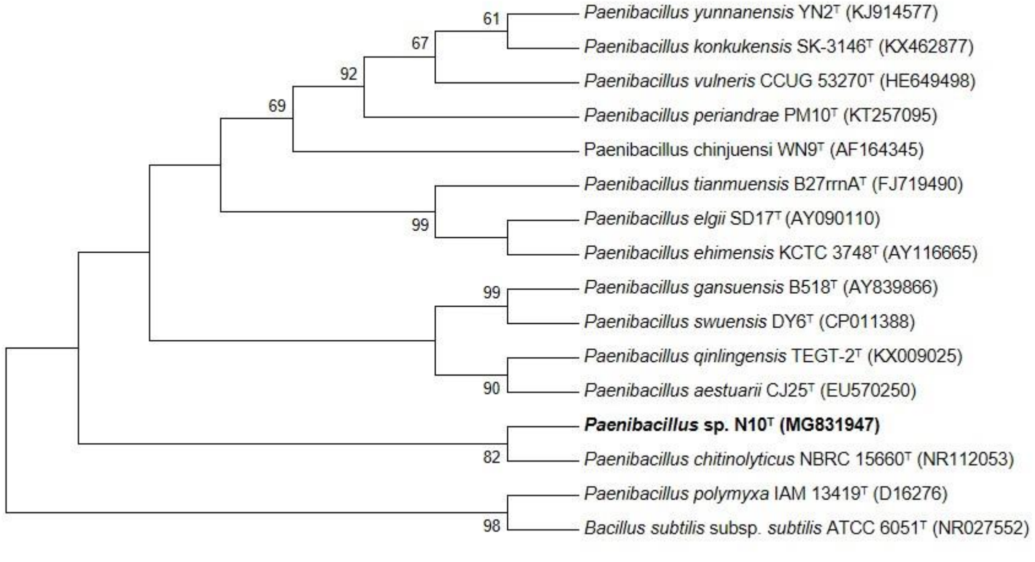

Figure S3: Molecular phylogenetic analysis of the 16S rRNA sequence using the maximum parsimony method. The most parsimonious tree (length = 592) is shown. The consistency, retention and composite indices are 0.501114, 0.554672 and 0.344796 (0.277954), respectively, for all sites and parsimony-informative sites (in parentheses). Bootstrap values are expressed as percentages of 1,000 replications, and those over 60% are shown at branch points. The *Bacillus subtilis* subsp. *subtilis* ATCC 6051^T sequence was used as the outgroup.

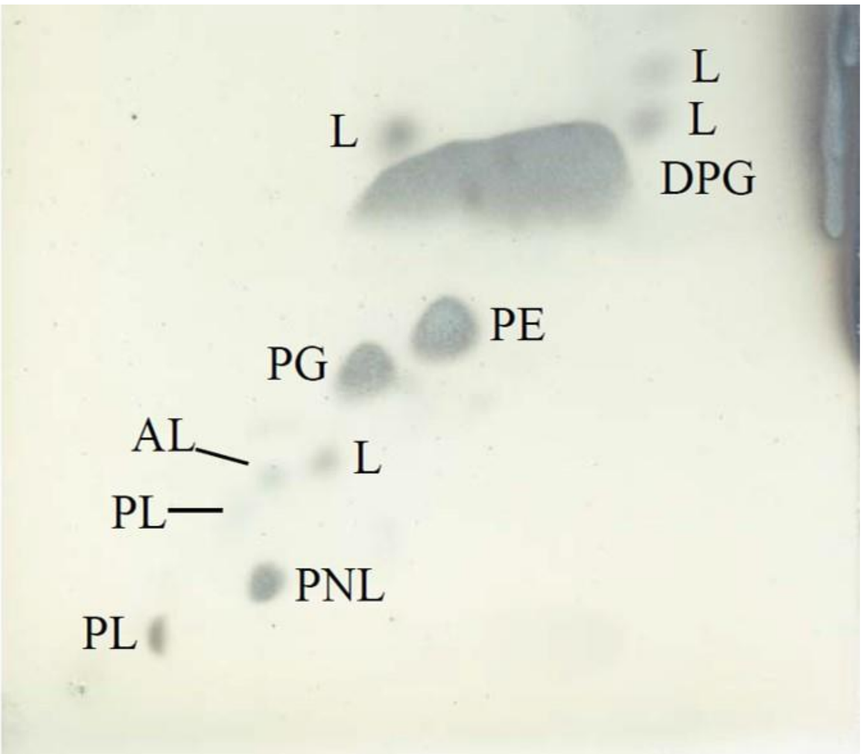

Figure S4: Polar lipid profile of strain N10^T determined after two-dimensional TLC using molybdatophosphoric acid. AL: aminolipid; DPG: diphosphatidylglycerol; L: lipid; PE: phosphatidylethanolamine; PG: phosphatidylglycerol; PL: phospholipid; PNL: phosphoaminolipid.

Table S1: ANIb and ANIm (in brackets) values among the genomes of strain N10^T (1) and the most related species of *Paenibacillus* genus: *P. chitinolyticus* LMG18047^T (2), *P. polymyxa* CECT155^T (3), *P. elgii* SD17^T (4), *P. vulneris* CCUG 53270^T (5) and *P. qinlingensis* TEGT-2^T (6).

Strains	1	2	3	4	5	6

1	-	74.42 [84.64]	66.94 [86.59]	68.41 [85.80]	68.57 [85.39]	68.00 [85.46]
2	74.43 [84.64]	-	66.93 [86.68]	69.22 [85.76]	68.48 [84.88]	67.91 [87.86]
3	67.71 [86.61]	67.81 [86.66]	-	67.29 [86.34]	67.68 [85.81]	67.72 [86.66]
4	68.20 [85.77]	69.01 [85.76]	66.83 [86.33]	-	70.47 [84.62]	67.30 [85.40]
5	68.16 [85.39]	68.19 [84.88]	66.88 [85.69]	70.34 [84.59]	-	67.86 [83.75]
6	67.29 [85.44]	67.33 [87.86]	66.35 [85.94]	66.85 [85.40]	67.42 [84.69]	-

Table S2: Genome sequence similarity between N10^{T} strain and genome sequences of closely related type strains with available genomes.

Strains	OrthoANI (%)	dDDH (%)
P. chitinolyticus LMG18047^{T}	75.60	21.1
P. polymyxa CECT155^{T}	68.40	31.4
P. elgii SD17^{T}	69.89	19.9
P. vulneris CCUG 53270^{T}	69.62	21.6
P. qinlingensis TEGT-2^{T}	69.19	19.3
